# Comparative selenoproteome analysis reveals a reduced utilization of selenium in parasitic platyhelminthes

**DOI:** 10.7717/peerj.202

**Published:** 2013-11-05

**Authors:** Liang Jiang, Hua-Zhang Zhu, Yin-Zhen Xu, Jia-Zuan Ni, Yan Zhang, Qiong Liu

**Affiliations:** 1 College of Life Sciences, Shenzhen University, Shenzhen, PR China; 2 College of Optoelectronic Engineering, Shenzhen University, Shenzhen, PR China; 3 Key Laboratory of Nutrition and Metabolism, Institute for Nutritional Sciences, Shanghai Institutes for Biological Sciences, Chinese Academy of Sciences, University of Chinese Academy of Sciences, Shanghai, PR China

**Keywords:** Selenocysteine, Parasite, Platyhelminthes, Selenoprotein, Bioinformatics

## Abstract

**Background.** The selenocysteine(Sec)-containing proteins, selenoproteins, are an important group of proteins present in all three kingdoms of life. Although the selenoproteomes of many organisms have been analyzed, systematic studies on selenoproteins in platyhelminthes are still lacking. Moreover, comparison of selenoproteomes between free-living and parasitic animals is rarely studied.

**Results.** In this study, three representative organisms (*Schmidtea mediterranea, Schistosoma japonicum* and *Taenia solium*) were selected for comparative analysis of selenoproteomes in Platyhelminthes. Using a SelGenAmic-based selenoprotein prediction algorithm, a total of 37 selenoprotein genes were identified in these organisms. The size of selenoproteomes and selenoprotein families were found to be associated with different lifestyles: free-living organisms have larger selenoproteome whereas parasitic lifestyle corresponds to reduced selenoproteomes. Five selenoproteins, SelT, Sel15, GPx, SPS2 and TR, were found to be present in all examined platyhelminthes as well as almost all sequenced animals, suggesting their essential role in metazoans. Finally, a new splicing form of SelW that lacked the first exon was found to be present in *S. japonicum*.

**Conclusions.** Our data provide a first glance into the selenoproteomes of organisms in the phylum Platyhelminthes and may help understand function and evolutionary dynamics of selenium utilization in diversified metazoans.

## Introduction

Selenium is an essential micronutrient for many organisms. It regulates a number of cellular processes in the form of selenocysteine (Sec) (Hatfield, 2001). Sec was first found in 1986 and has been known as the 21st amino acid. It is cotranslationally incorporated into selenium-containing proteins (or named selenoproteins) in response to the codon UGA, which normally functions as a stop codon. This process needs both a Sec insertion sequence (SECIS) element and several *trans*-acting factors (Hatfield, 2001). To date, twenty-five and twenty-four selenoproteins have been characterized in human and mouse, respectively (Kryukov et al., 2003). The functions of only about half of them have been identified, most of which are important enzymes related to energy metabolism or antioxidant process, such as glutathione peroxidases (GPxes), thioredoxin reductases (TRs) and deiodinases (DI) (Atkins & Gesteland, 2000; Bock, 2000; Kryukov, Kryukov & Gladyshev, 1999).

In recent years, an increasing number of genome sequencing projects have provided a large volume of gene and protein sequence information. Several bioinformatics tools have been developed to identify the whole set of selenoproteins (or selenoproteome) that one organism may have (Kryukov et al., 2003; Korotkov et al., 2002). These programs have successfully identified many new selenoproteins in bacteria, algae, insects, nematodes, and a variety of both invertebrates and vertebrates, implying that selenium plays an important role in all the three kingdoms of life (Castellano et al., 2001; Kryukov & Gladyshev, 2000; Lobanov et al., 2006a; Lobanov et al., 2006b; Mariotti et al., 2013; Mariotti et al., 2012; Novoselov et al., 2002; Zhang, Fomenko & Gladyshev, 2005; Zhang & Gladyshev, 2008; Zhang et al., 2006). It is obvious that investigation of the selenoproteomes of various organisms is helpful for understanding evolutionary dynamics of selenium utilization as well as its relationship with different living conditions and lifestyles.

Recently, we have analyzed the selenoproteomes in a subset of newly sequenced organisms to investigate the evolutionary trends of different selenoprotein families in metazoan by using our SelGenAmic algorithm (Jiang, Liu & Ni, 2010; Jiang, Ni & Liu, 2012). These studies uncovered new features for selenium utilization, which suggested that only a few selenoproteins have evolved or lost throughout metazoan history. Massive loss events of selenoproteins were only detected in isolated evolutionary branches such as nematodes and insects, both of which belong to ecdysozoa. Ecdysozoa is a sub-phylum of protostomia; however, it is unclear if similar events occurred in other sub-phyla of protostomia, such as platyhelminthes.

Platyhelminthes are bilaterally symmetrical animals, which have three main cell layers, while the more primitive radially symmetrical cnidarians and ctenophores (comb jellies) have only two cell layers. In addition, unlike other bilaterians, platyhelminthes have neither internal body cavity nor specialized circulatory and respiratory organs. These features limit platyhelminthes to sizes and shapes that enable oxygen to reach and carbon dioxide to leave all parts of their bodies by simple diffusion (Walker & Anderson, 2001; Hinde, 1998; Ruppert, Fox & Barnes, 2004).

In this study, we reported an advanced analysis of the selenoproteomes of several animals of the phylum Platyhelminthes based on the SelGenAmic algorithm. We chose *Schmidtea mediterranea, Schistosoma japonicum* and *Taenia solium* as representatives of three sub-phyla of Platyhelminthes: Rhabditophora, Trematoda and Cestoda, respectively. These organisms have different living environments and lifestyles. *S. mediterranea* is a free-living planarian and has become a model for regeneration, tissue development and stem cell biology (Robb, Ross & Sanchez Alvarado, 2008). *S. japonicum* and *T. solium* are important eukaryotic parasites, which live in the organs of hosts and cause harmful parasitosis of humans and their livestock (Schistosoma japonicum Genome & Functional Analysis, 2009; Tsai et al., 2013). We detected all known selenoprotein genes in these organisms. Only a small number of selenoproteins were present in all three examined platyhelminthes, implying they play an essential role for these organisms with different lifestyles. It appeared that parasitic lifestyle may reduce the selenoproteome in both size and families. Interestingly, the first exon of selenoprotein W (SelW) gene was absent in *S. japonicum*, resulting in the presence of a new form of SelW in this organism. Thus, our results provide new insights into understanding evolution and function of selenoproteins in metazoans.

## Materials & Methods

### Data resources

The genome and expressed sequence tag (EST) sequences of *S. mediterranea* and *S. japonicum* were downloaded from NCBI, the data of *T. solium* were downloaded from the website of the *T. solium* Genome Project at National University of Mexico (UNAM) [ftp://bioinformatica.biomedicas.unam.mx/]. Information about the statistics of both genomes and ESTs of these organisms is shown in Table 1.

**Table 1:** Summary statistics of data resources.

Organism	Genome size (Mbp)	Number of contigs	EST size (Mbp)	Number of ESTs
*S. mediterranea*	865	94,682	61	78,678
*S. japonicum*	369	95,265	75	103,874
*T. solium*	118	5,508	50	74,427

### General procedure for selenoprotein identification

The overall strategy was similar to that previously described (Jiang, Liu & Ni, 2010; Jiang, Ni & Liu, 2012). In general, the whole genome sequences were scanned to find all TGA codons and all exons containing in-frame TGA codons. Selenoprotein gene candidates were built by using SelGenAmic algorithm (Jiang, Liu & Ni, 2010). These candidates were further screened with conservation in the local regions flanking the TGA codon. EST resources were used to help build correct gene structures of selenoprotein genes. The 3′ untranslated region of each gene was checked for the presence of SECIS element. Details are shown as follows.

### Construction of ORFs containing Sec-TGAs

A series of perl scripts were developed to obtain TGA codons from the genome and build TGA containing exons from common signals and TGA codons. A perl program was developed based on the SelGenAmic algorithm to construct all genes containing in-frame TGA codons. The SelGenAmic algorithm is used to find an optimal ORF for each exon containing TGA codon. Here, the word “optimal” means that the coding potential score of such ORF is larger than other ORFs composed of TGA-containing exon and other suitable normal exons. Based on dynamic programming, all optimal ORFs for TGA-containing exons can be built in linear time. More details of the SelGenAmic algorithm have been described in our previous study (Jiang, Liu & Ni, 2010).

### Homology analysis and multiple alignment

All genes containing in-frame TGA codon were searched by the program BLASTP (version 2.2.18) (Altschul et al., 1997) with an E-value cut-off at 1. All similar sequences detected were used to create multiple sequence alignments with ClustalW (version 1.83) (Thompson, Higgins & Gibson, 1994). The conservative motif containing the Sec residue of any gene was analysed by the program using a motif search algorithm like MAME.

### Search for SECIS elements

The SECIS element patterns used in this study are the same as those used to search for metazoan SECIS elements. The COVE score of SECIS-like structures were evaluated by the online program SECISearch (version 2.19) (Korotkov et al., 2002; Kryukov et al., 2003).

### Gene structure analysis

EST sequences were compared with all predicted selenoprotein genes using the program BLASTN with an E-value cut-off at 0.001. Highly similar EST sequences were spliced using the SeqMan program from the DNASTAR package [http://www.dnastar.com/] and analyzed for identification of gene structures. The constructed genes were compared to genomic sequences with the program Sim4 (Florea et al., 1998) to find the locations of exons and introns in the genome, shown as position numbers in gene structure figures.

## Results and Discussion

### Identification of selenoprteins in platyhelminthes genomes

In the recent decade, computational prediction of selenoprotein genes in genomic database has become a major strategy for understanding the important roles of selenium. Based on the specific selenocysteine insertion and selenoprotein biosynthesis mechanisms (such as UGA codon, SECIS element, etc.), as well as the conservation of the sequence that flanks selenocysteine in different homologs of selenoproteins, several bioinformatics algorithms, such as SECISearch and SelGenAmic, have been developed to identify new selenoproteins in a variety of organisms including human (Castellano et al., 2001; Jiang, Liu & Ni, 2010; Jiang, Ni & Liu, 2012; Kryukov et al., 2003; Kryukov, Kryukov & Gladyshev, 1999; Mariotti et al., 2013; Zhang & Gladyshev, 2008; Zhang et al., 2006). Many of these newly predicted selenoproteins have been experimentally verified, implying that these computational approaches are highly efficient for selenoprotein identification. Recent comparative genomics analyses also revealed that many of these selenoproteins are widespread in many eukaryotes including both vertebrates and invertebrates (Jiang, Ni & Liu, 2012; Mariotti et al., 2012).

In this work, to identify all selenoprotein genes in the genomic dataset of three platyhelminthes (*S. mediterranea, S. japonicum* and *T. solium*), we employed a strategy based on the SelGenAmic algorithm, which have been used to successfully identify the selenoproteomes of several invertebrates such as *Nematostella vectensis* and *Lottia gigantea* (Jiang, Liu & Ni, 2010; Jiang, Ni & Liu, 2012). The number and family of selenoproteins found in each organism are shown in Table 2. A total of 37 selenoprotein genes have been identified; all of them are members of known selenoprotein families.

**Table 2:** Selenoproteins identified in platyhelminthes. The numbers indicate how many proteins were identified in each selenoprotein family by organism.

	SelT	Sel15	GPx	SPS2	TR	SelK	SelW	SelO	SelU	SelM	SelS	Total
Sm	1	1	2	2	2	1	6	1	1	1	1	19
Sj	1	1	3	2	1	1	2					11
Ts	1	1	3	1	1							7

**Notes.**

Organisms are represented by abbreviations Sm
*Schmidtea mediterranea*
Sj
*Schistosoma japonicum*
Ts
*Taenia solium*

Selenoprotein name abbreviations SelT selenoprotein T Sel15 15kD selenoprotein GPx glutathione peroxidase SPS2 selenophosphate synthetase 2 TR thioredoxin reductase SelK selenoprotein K SelW selenoprotein W SelO selenoprotein O SelU selenoprotein U SelM selenoprotein M SelS selenoprotein S

*S. mediterranea* is a free-living, freshwater planarian that lives in southern Europe and Tunisia (Lazaro et al., 2011; Harrath et al., 2004). It is a model organism for the research of regeneration and development of tissues such as the brain and germline (Reddien & Sanchez Alvarado, 2004; Salo & Baguna, 2002). Here, a total of 19 selenoprotein genes, which belong to 11 previously described selenoprotein families, were identified. Compared to other invertebrates, especially some basal group of multicellular animals such as sponge (22 selenoproteins belonging to 16 selenoprotein families) and *Trichoplax* (28 selenoproteins belonging to 13 selenoprotein families) (Jiang, Ni & Liu, 2012), the selenoproteome of *S. mediterranea* is quite similar to them, implying that the ancestor of *S. mediterranea* or rhabditophora, or even that of platyhelminthes, have remained almost all selenoproteins from the common ancestor of metazoans.

Compared to *S. mediterranea*, two parasitic platyhelminthes, *S. japonicum* and *T. solium* have significantly reduced selenoproteomes at the level of both size and selenoprotein families. *S. japonicum* is an important eukaryotic parasite and one of the major infectious agents of schistosomiasis, a disease that still remains a significant health problem especially in lake and marshland regions. Moreover, *S. japonicum* is the only human blood fluke that occurs in China. Approximately one million people in China, and more than 1.7 million bovines and other mammals, are currently infected (Zhou et al., 2005). Using our approach, eleven selenoproteins belonging to 7 families were detected in *S. japonicum*, suggesting selenoprotein loss events in this organism or in the sub-phylum Trematoda.

Similarly, only seven selenoprotein genes that belong to 5 families were found in *T. solium* (pork tapeworm). About 50 million people worldwide and millions of their porcine livestock become infected with this parasite. Neurocysticercosis resulting from penetration of *T. solium* larvae into the central nervous system is a major cause of acquired seizures in human (White, 1997). Our data suggest that massive loss events of selenoprotein genes occurred in the sub-phylum Cestoda of platyhelminthes.

It should be noted that although both *S. mediterranea* and *T. solium* are parasites, degrees of their dependence on hosts are different. The life cycle of *S. japonicum* is complicated and two free-swimming larva stages (miracidia and cercaria) are indispensable. Once the cercaria penetrates the skin of the host it becomes a schistosomule, the adult worms living at the mesenteric veins. Thus, *S. japonicum* could be somewhat considered as a “incomplete parasite” or half-parasite. In contrast, no free-living stage was found in the life cycle of *T. solium*. Thus, *T. solium* was considered to have a higher-level parasitic degree than *S. japonicum*, or “complete parasite”.

### Comparative analysis of platyhelminthes selenoproteomes

**Figure 1:**
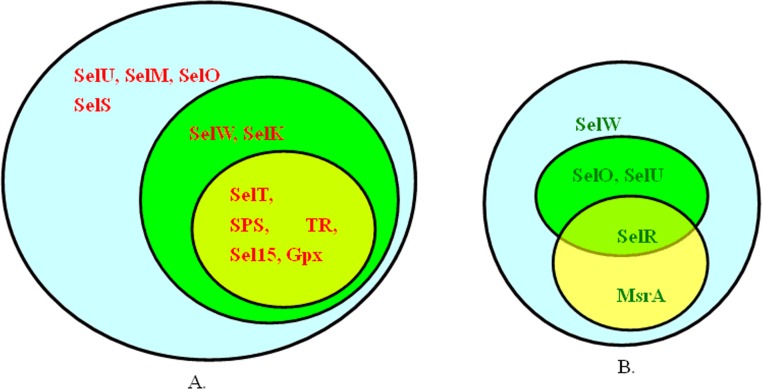
Comparison of selenoproteomes in platyhelminthes. (A) The outer blue cycle includes all the selenoproteins found in *S. mediterranea*, the intermediate green cycle includes all the selenoproteins found in *S. japonicum*, and the inner yellow cycle includes all the selenoproteins found in *T. solium*. (B) Proteins in these cycles are Cys-containing homologs of selenoproteins, in which the Sec is replaced with Cys residue. Blue cycle: *S. mediterranea*; green cycle: *S. japonicum*; yellow cycle: *T. solium*.

Comparison of selenoproteomes of the three platyhelminthes revealed that selenoproteomes reduced gradually at the level of size and selenoprotein families according to the order from *S. mediterranea* (free-living) to *S. japonicum* (incomplete parasite) to *T. solium* (complete parasite). As shown in Fig. 1, the number of selenoprotein families of *S. mediterranea* is much more than that of the other two platyhelminthes. All selenoprotein families in *S. japonicum* and *T. solium* are present in *S. mediterranea*, and all selenoprotein families in *T. solium* are found as a subset of those in *S. japonicum*.

First, five selenoprotein families (SelT, SPS2, TR, Sel15 and GPx) were found to be present in all the three organisms, implying that these selenoproteins might be essential for all platyhelminthes regardless of their lifestyles. The functions of four selenoprotein families have been thoroughly documented by other research groups. Selenophosphate synthetase 2 (SPS2) is an essential component of Sec biosynthesis (Turanov et al., 2011). Thioredoxin reductases (TR) are a group of enzymes known to catalyze the reduction of thioredoxin and hence are central components in the thioredoxin system (Lee, Kim & Lee, 2013). The 15kD selenoprotein (Sel15) has redox function and may be involved in the quality control of protein folding (Kasaikina et al., 2011). Glutathione peroxidises (GPxes) are important enzymes with peroxidase activity, whose major biological role is to protect the organism against oxidative damage (Bhabak & Mugesh, 2010). The function of the fifth selenoprotein, selenoprotein T (SelT), is not clear so far. It was reported that SelT was highly induced in endocrine and metabolic tissues during ontogenetic and regenerative processes (Tanguy et al., 2011). Thus, it could be a potential target to investigate the mechanism of regeneration and development of planarian, as well as the relationship with the longevity of parasites (Collins et al., 2013).

Four *S. mediterranea* selenoprotein families (SelU, SelM, SelO and SelS) have not been detected in either *S. japonicum* or *T. solium* selenoproteome. Among them, SelM and SelS appeared to have been completely lost, suggesting that these two selenoproteins are not needed by parasitic platyhelminthes. On the other hand, SelO and SelU are replaced with cysteine(Cys)-containing homologs in these two organisms. It is possible that although functions of these two proteins are still needed for parasitic platyhelminthes, the ability to use selenium decreases and Sec residue has been replaced by Cys. In addition, all selenoprotein genes detected in *T. solium* were also found in *S. japonicum*, while two *S. japonicum* selenoproteins, SelW and selenoprotein K (SelK), have lost in *T. solium*. It appeared that the size of selenoproteomes in platyhelminthes is associated with the lifestyles. The free-living animal *S. mediterranea* has the largest selenoproteome, the “incomplete parasite” *S. japonicum* has smaller one and the “complete parasite” *T. solium* has the smallest selenoproteome among them. It should be noted that a partial SelW-like sequence was found in the genomic and EST sequences of *T. solium*; however, its N-terminal region containing the Sec codon is missing. In this study, we did not consider it as a selenoprotein gene. Further studies are needed when additional data are available for this organism.

The above results suggested that, free-living organisms have larger selenoproteome whereas parasites have reduced selenoproteome. Previously, the selenoproteomes of several parasitic protozoans, including *Plasmodium*, *Trypanosoma* and *Leishmania* species, were reported. Similar to the parasitic platyhelminth, few selenoproteins were identified in these parasites: 4 selenoproteins were found in *plasmodium*, and 2 or 3 selenoproteins in *Trypanosoma* and *Leishmania* (Lobanov et al., 2006a; Lobanov et al., 2006b). These data are consistent with what we observed in this work, suggesting that a parasitic lifestyle may be an important factor that leads to the reduction of selenium utilization.

### Comparison of platyhelminthes selenoproteomes with other animals

**Figure 2:**
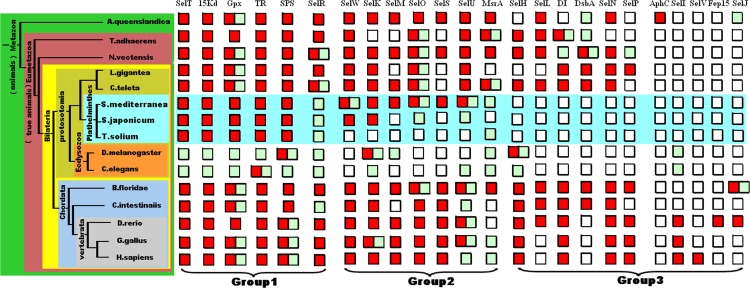
Distribution of selenoproteins in different metazoan taxa. Selected branches of animals are shown in the phylogenetic tree on the left. Different selenoprotein families are shown on the top. Red boxes indicate the existence of selenoproteins in one organism. Green boxes indicate the existence of Cys-containing homologs of selenoproteins. Blank boxes indicate that neither selenoprotein nor Cyscontaining homologs is detected. The selenoproteomes of platyhelminthes are highlighted light blue.

Figure 2 shows the distribution of selenoproteins and their Cys-containing homologs in different taxa of the animal kingdom, including platyhelminthes and other animals. The schematic phylogenetic tree and selenoprotein map were built based on our earlier study on the evolution of metazoan selenoproteins (Jiang, Ni & Liu, 2012). Comparison of the selenoproteomes between platyhelminthes and ecdysozoa revealed that, massive loss of selenoproteins did not happen in the early age of platyhelminthes phylum (19 selenoproteins found in the planarian *S. mediterranea*). However, several selenoprotein loss events did occur when the lifestyle of platyhelminthes changed from free-living to parasitic stages.

We divided all known animal selenoprotein families into three groups. The first group contains the five selenoproteins detected in all examined platyhelminthes (SelT, Sel15, Gpx, TR and SPS) as well as SelR. It is obvious that these five selenoproteins are widespread in many other phyla of animals, including platyhelminthes. In addition, although massive selenoprotein loss events have been reported in ecdysozoa (insects and nematodes), all these selenoprotein families are either retained or replaced with Cys-containing homologs, implying that their functions are essential for all animals even if selenium is not used. Thus, we considered them to be the most indispensable selenoproteins in animals. Another selenoprotein family, SelR, is also included in this group because SelR is also reported to be present in many phyla of animals, especially all vertebrates (Mariotti et al., 2012). However, the fact that only Cys-containing homologs were found in, platyhelminthes, implying that it may have become selenium-independent in this phylum.

The second group contains 7 selenoprotein families: SelM, SelO, SelS, SelU, methionine sulfoxide reductase A (MsrA), SelK and SelW, which are present in free-living planarians but have massively lost in parasites. Except ecdysozoa, these selenoproteins are generally conserved in other phyla. The loss of these selenoproteins in parasitic platyhelminthes may suggest that they only play important roles in free-living lifestyle.

All the selenoproteins in the third group are totally lost in these platyhelminthes and no Cys-containing homologs could be detected. Some selenoproteins have been known to be present in specific phyla, such as Fep 15 in fish, SelJ in fish and amphioxus, SelI in vertebrates, SelV in plancentals and AphC-like selenoprotein in sponge. These selenoproteins most likely evolved recently in certain phylum. Occurrence of other selenoproteins in this group is much more extensive. For example, selenoprotein P (SelP) contains multiple Sec residues (up to 17 Sec residues in zebra fish), which is considered to play an important role in the preservation and transport of selenium. Other selenoproteins, SelH, SelL, SelN, DI and DsbA are found in many other animals. The loss of these selenoproteins in platyhelminthes implied that they might have lost before the split of platyhelminthes.

### Analysis of SelW genes

SelW is widespread in all three kingdoms of life. A previous metagenomic analysis of microbial selenoproteomes showed that SelW is one of the most abundant selenoprotein genes in the ocean microbes (Zhang & Gladyshev, 2008). The biological function of SelW has not been identified. It can serve as an antioxidant, responds to stress, involved in cell immunity, specific target for methylmercury, and has thioredoxin-like function (Whanger, 2009).

Several SelW genes were found in platyhelminthes. Six SelW genes were found in *S. mediterranea*. Three of them belong to the SelW1 sub-family (named Sm.SelW1_a, Sm.SelW1_b and Sm.SelW1_c). The other three (Sm.SelW2_a, Sm.SelW2_b and Sm.SelW2_c) belong to the SelW2 sub-family. Besides, a Cys-containing homolog of SelW (Sm.SelW.Cys) was also present in this organism. As shown in Fig. 5, Sm.SelW.Cys is similar to the bacterial SelW in *Alkaliphilus metalliredigenes*. The number of SelW genes in *S. mediterranea* is three times of that in *S. japonicum*, which only possessed two SelW genes, Sj.SelW1 and Sj.SelW2. Only a partial SelW-like sequence could be found in *T. solium* (Ts.SelW). Similar to the trend of selenoproteome size, the number of SelW genes is also associated with the lifestyles of these organisms: free-living > incomplete parasite > complete parasite.

**Figure 3:**
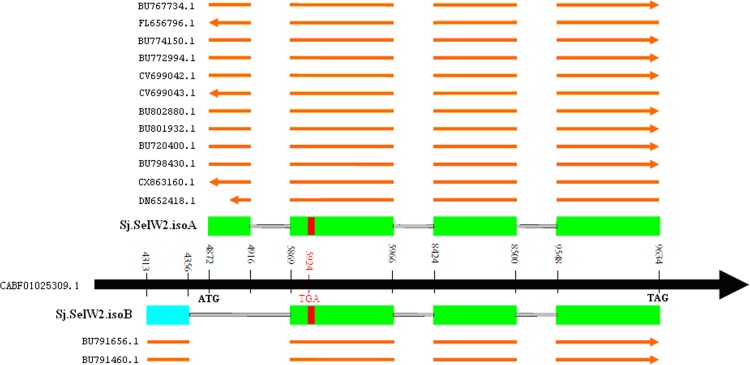
Two isoforms of *S. japonicum* SelW2 genes. The genome sequence is indicated by a black arrow-line. The coding exons are indicated with green boxes. The un-coding exon in isoformB is indicated with a blue box. EST sequences are indicated with the orange arrow-line. The start codon, stop codon and TGA-Sec codon are highlighted. The accession number of genomic scaffold sequence and EST sequences as well as their locations on the scaffold are shown.

Besides the regular transcript of SelW2 gene (named Sj.SelW2.isoA), a new splicing isoform (named Sj.SelW2.isoB) was detected in *S. japonicum*. The most relevant difference between these two isoforms is that the first exon of Sj.SelW2.isoA is not present in Sj.SelW2.isoB (shown in Figs. 3 and 5). The Sj.SelW2 gene is found in the scaffold CABF01025309. The Sj.SelW2.isoA splicing form has a complete SelW open reading frame (ORF) which was supported by 12 EST sequences. The Sj.SelW2.isoB form was supported by 2 EST sequences. It is possible that this new isoform is mis-annotated by sequencing error or genomic sequence contamination.

**Figure 4:**
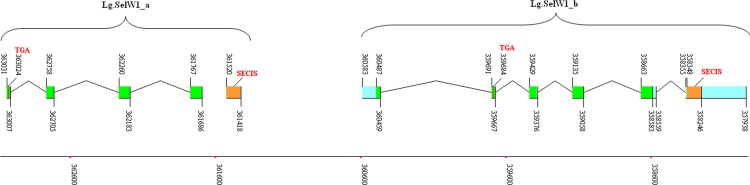
Gene structure and locations of Lg.SelW1_a and Lg.SelW2_b. The coding regions are indicated by green rectangles, the untranslated regions by blue rectangles, and the SECIS elements by orange rectangles. An intron is indicated by lines connecting the exons. The position of each site in the sequence of the chromosome of scaffold is shown by numbers and bottom coordinates. The Sec-TGA codon is highlighted by the red words.

**Figure 5:**
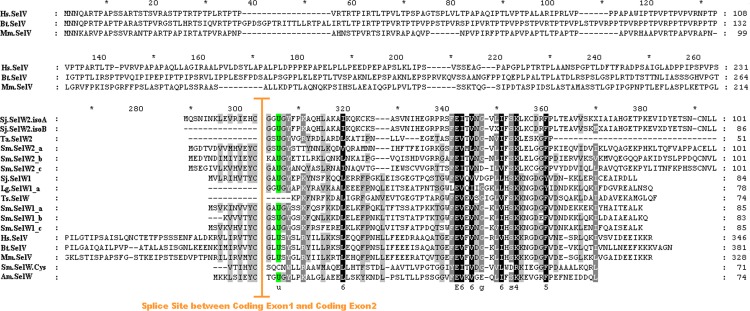
Multiple alignments of SelW proteins. Sec residues are highlighted green. The splice site between the first coding Exon and the second coding Exon in SelW family were shown with an orange vertical line. Organisms are represented by abbreviations: Sm, *Schmidtea mediterranea*; Sj, *Schistosoma japonicum*; Ts, *Taenia solium*; Lg, *Lottia gigantean*; Ta, *Trichoplax adhaerens*; Hs, *Homo sapiens*; Bt, *Bos taurus*; Mm, *Mus musculus*; Am, *Alkaliphilus metalliredigenes*.

To further study the possibility of a new splicing form of SelW gene, we analyzed all sequenced eukaryotes and found additional evidence for the presence of this new isoform. A SelW1 gene (named Lg.SelW1_a) found in *Lottia gigantean* also lacked the first exon (Figs. 4 and 5). Notably, another SelW1 gene (Lg.SelW1_b) is located downstream of Lg.SelW1_a. Similar exon composition and sequence homology were found between them, implying a recent gene duplication of SelW in this organism. In addition, a SelW2 gene (Ta.SelW2) was found in *Trichoplax adhaerens*, which also lacked the first exon (Fig. 5). The exon-intron organizations of these invertebrate SelW genes have been reported in our previous work which focused on the evolution of metazoan selenoproteomes (Jiang, Liu & Ni, 2010). The existence of Lg.SelW1_a and Ta.SelW2 suggested that the first exon of SelW genes may have lost in certain organisms, probably because of unexpected gene duplication and recombination events. Further efforts are needed to identify the complete sequence of this new SelW isoform.

## Conclusions

In this study, we report a comprehensive analysis of the selenoproteomes of three representative organisms in the phylum Platyhelminthes. We found that the size of selenoproteomes and corresponding selenoprotein families are related to different lifestyles. Free-living organisms have larger selenoproteome whereas parasites, especially “complete parasite”, have reduced selenoproteomes. Comparison of the selenoproteomes between platyhelminthes and other animals revealed that the ancestor of platyhelminthes have remained almost all selenoproteins from the common ancestor of metazoans. A new SelW isoform was found to be present in *S. japonicum*, which lack the first exon of SelW gene. Further studies will be needed to test this new isoform and identify additional factors that influence selenium utilization.
